# Odor perception of aromatherapy essential oils with different chemical types: Influence of gender and two cultural characteristics

**DOI:** 10.3389/fpsyg.2022.998612

**Published:** 2022-11-10

**Authors:** Jie Chen, Nan Zhang, Shichun Pei, Lei Yao

**Affiliations:** ^1^School of Design, Shanghai Jiao Tong University, Shanghai, China; ^2^Aromatic Plant R&D Center, Shanghai Jiao Tong University, Shanghai, China

**Keywords:** odor perception, affective responses, aromatherapy essential oils, characteristics, region, fragrance usage habits

## Abstract

Olfactory perception, and especially affective responses of odors, is highly flexible, but some mechanisms involved in this flexibility remain to be elucidated. This study investigated the odor perceptions of several essential oils used in aromatherapy with emotion regulation functions among college students. The influences of people’s characteristics including gender, hometown region, and fragrance usage habit on odor perception were further discussed. Odor perception of nine essential oils, which can be divided into the ester-alcohol type (e.g., lavender oil) and terpene type (e.g., lemon oil) were evaluated under three odor concentrations. The results indicated that chemical type, but not concentration, significantly influenced the odor perception and there was no interaction between the two factors in this study. The arousal and emotional perception scores of odors with terpene-type oil were significantly higher than odors with ester-alcohol type. In terms of people’s characteristics, participants from the southern Yangtze river gave a higher familiarity rating to almost all of these odors. The habits of fragrance usage also significantly influenced some of the odors’ subjective intensity and emotional perception ratings. However, there were no significant gender differences in most of the odor perceptions. In addition, familiarity and pleasantness were positively correlated, and emotional perception and subjective intensity also showed a weak correlation. These results suggested that users’ cultural characteristics could be considered to be important factors that affect the essential oil’s odor perception in aromatherapy.

## Introduction

Olfactory perception is known to be highly flexible and is related to the age, gender, cultural background of the perceiver, the environment in which the odor is perceived or the characteristics of the odorant itself like its chemical composition or its concentration. The olfactory (Delplanque et al., 2008) environments influence people’s emotions and the connection between olfaction and emotion is particularly close. In the olfactory process, odor molecules enter the nasal cavity and attach to the cilia of olfactory receptors in the olfactory epithelium (Mackay-Sim et al., 2006). Then the guanine nucleotide-binding protein (G-protein) coupled receptors are activated and electrical signals are generated. Electrical signals are then transmitted to the brain *via* the olfactory bulb and higher olfactory cortex by olfactory sensory neurons (Sell, 2006; Angelucci et al., 2014). These electrical signals further affect the limbic system, which is closely related to emotion regulation (Laurent and Gilles, 2002). Therefore, essential oils, perfumes and incenses have been used for self-adornment, and modification of the living environment since ancient times.

In recent years, essential oils have been increasingly used to improve people’s olfactory environment for their naturalness and possible efficacy in improving mood. A study of older adults found that after inhaling drops of 1.5% lavender oils for 30 nights, statistically significant improvement occurred in the scores of depression, anxiety, and stress-scale (Ebrahimi et al., 2021). Inhalation of bergamot oil was also found to reduce the salivary alpha-amylase level and scores on the state–trait anxiety inventory (Watanabe et al., 2015). However, aromachology research has found that odor subjective perception was relevant to the possible impact of odor (Herz, 2009). For example, the degree of odor pleasantness would affect the emotional changes of the subjects (Villemure et al., 2003; Burnett et al., 2004). Essential oils are composed of various volatile chemical components, which are mainly classified as terpenes, esters, alcohols, etc. The constituent differences lead to the aroma type differences. Meanwhile, due to individual differences, people have different perceptions of odors, which may influence the potential effects of those functional odors.

Many factors can affect odor sensory evaluation. In terms of the odor itself, both type and concentration are important. Odor classification relies mainly on the classification of objects as odorant sources (Dubois, 2000), such as floral odor, fruit odor, peppermint odor, etc. Ba et al. (Ba and Kang, 2019) found that the mean scores of olfactory comfort and odor familiarity for food odors were higher than those for plant odors, and both elevated with increased concentration. However, the scores of the subjective intensity of different odors at three concentrations did not differ significantly in this study. Odors can also be classified according to their chemical composition. As we all know, most of the odors in the environment are made up of various monomeric compounds, with terpenes, esters, and alcohols being the main categories. Of the ten most common single compounds in floral odor identified so far, five are terpenes (limonene, etc.), three are alcohols (linalool, etc.), and one is an ester (methyl salicylate; Knudsen et al., 2006). The terpene-type odors and the ester-alcohol type odors also play an important role in food odors, which seem to comprise a category of particular importance to humans. A study on the odorant hedonic value of 23 monomeric compounds found that isoamyl acetate and geraniol had higher pleasantness and familiarity scores than limonene, while limonene had a relatively low score of subjective intensity among all compounds (Chalencon et al., 2022).

The odor information like verbal labels also influences people’s judgment of odors (Sorokowska et al., 2015a), negative labels had no effect on intensity ratings but would affect the subject’s preference for the odors (Zellner et al., 2014). Some studies have confirmed the existence of the halo effect of natural ingredient claims (Apaolaza et al., 2014). In addition, the pleasantness ratings of odors were found to be modulated by the knowledge of their identity due to prior experience and this relationship might be more evident in unpleasant odors (Martinec Novakova et al., 2015).

In terms of individual differences, biological makeup, personal experience, and the environment have also been shown to influence odor sensory evaluation (Majid et al., 2017). There is tremendous variation within and between populations in olfactory receptor genes. Some specific genes may be linked to the olfactory ability associated with particular odors (Li et al., 2022). In addition, gender is an important determinant of the ability to identify odors (Bontempi et al., 2021). Women are often considered to have better olfactory abilities than men (Larsson et al., 2003; Greenberg et al., 2013) and they are generally more attentive to odors (Ferdenzi et al., 2008). However, it has also been suggested that the superiority of women in odor detection ability may be only for specific odors. For example, Thriel et al. found only the odor thresholds of trimethylamine were significantly affected by gender, while all other odor thresholds were not affected (Van Thriel et al., 2008). Some studies have even shown that men are better at detecting specific odors (Olsson and Laska, 2010). Personal experiences such as age, mere exposure to odors (Schriever et al., 2014), etc. were also associated with odor identification abilities. Most studies show that human olfactory function peaks in adulthood and declines with age (Sorokowska et al., 2015b). It was discovered that seniors had the same ability to identify unpleasant odors, whereas the identification of pleasant odors was decreased among seniors when compared to young adults (Joussain et al., 2013). In addition to the factors mentioned above, there appear to be variances in odor perception among people in different environments, as environmental differences bring with them a range of differences in climate, vegetation conditions, dietary habits, and culture. Factors such as ethnic background (Ayabe-Kanamura et al., 1998; Sorokowska et al., 2015c) were also associated with odor identification abilities. But ethnicity did not seem to influence the evaluation of odor intensity or the distribution of mood responses. A study comparing the ability of subjects from Japan and the Netherlands to detect m-xylene odors reported a 10-fold difference in chemical identification between the groups (Hoshika et al., 1993). Jin et al. found that Caucasian participants preferred cinnamaldehyde more than East Asian participants (Jin et al., 2018). At present, the research objects of related research mainly focus on food odor or monomeric compound odor, and there is relatively little research on the sensory evaluation of functional odor combined with chemical types.

Therefore, the aims of this study were: (1) to understand the affective responses of essential oil odors with healthy function at different concentrations, (2) to understand the influence of participants’ characteristics on odor sensory evaluation, and (3) to understand the correlation between the odor evaluation indexes. According to the chemical constituent and function, six essential oil odors which were commonly used in the mainstream aromatherapy market for anxiolytic or antidepressant treatment and their compound odors were used in this study, and young healthy adults with different gender, regional culture, and living habits were selected to evaluate the odor subjective indexes.

## Materials and methods

### Participants

*A priori* power analysis was selected from the F test family in G*Power 3.1.7 software (Heinrich Heine, Universität Düsseldorf) for sample size estimation. The effect size was assumed to be 0.25, α err prob. to 0.01, and power (1 - β err prob) to 0.95, and the total sample required was estimated to be at least 28. Fifty healthy students (25 females and 25 males) including 40% undergraduates, 56% postgraduates, and 4% doctoral students, with an average age of 22 (SD = 2.6; Min = 18; Max = 29), with self-reported normal olfaction, no mental illness, no rhinitis or other olfactory disorders and not pregnant, were recruited for the experiment. The study was performed in accordance with the Declaration of Helsinki on Biomedical Research involving human subjects and approved by the Research and Ethics Offices of the Shanghai Jiao Tong University (NO.H2022015I). All participants were recruited from Shanghai Jiao Tong University through networking contacts. The privacy rights of participants always be observed.

### Odor preparation

Nine essential oils were used for the preparation of the odor samples, including six essential oils [lavender oil (*Lavandula angustifolia*, LVO), clary sage oil (*Salvia sclarea*, CSO), bergamot oil (*Citrus × bergamia*, BGO), lemon oil (*Citrus × limon*, LMO), rosemary oil (*Rosmarinus officinalis*, RMO), copaiba oil (*Copaifera officinalis*, CPO)], and three blend essential oils. The essential oils used in this study were sourced from the Aromatic Plant Research and Development Centre at Shanghai Jiao Tong University. Blended oil-I (blended-I) was prepared from the six essential oils mentioned above according to the volume ratio of 1:1:1:1:1:1; blended oil-II (blended-II) was composed of LMO, RMO, and CPO according to the volume ratio of 1:1:1; blended oil-III (blended-III) was composed of LVO, CSO, and BGO according to the volume ratio of 1:1:1. These essential oils have been selected based on both chemical constituents and efficacy. Firstly, the main constituents of LVO, CSO, and BGO are esters and alcohols; the main constituents of LMO, RMO, and CPO are terpenes. Secondly, the selected oils have a range of bioactive properties (e.g., antibacterial (Wang et al., 2012; Ojeda-Sana et al., 2013; Ontas et al., 2016), anti-inflammatory (Lucca et al., 2018; Pandur et al., 2021), etc.), and also have been proved to alleviate emotional disorders (Yoshizawa et al., 2015; Samadi et al., 2021), so they have a broad application prospect.

An aromatherapy machine that using air pressurized atomization technology was used to atomize the essential oil into the 500 ml olfactory bottle to prepare the odor sample for evaluation. The odor samples were set to low, medium, and high intensity groups, in which the essential oils were atomized into the bottle for 3, 15, and 75 s, respectively. According to the measurement results, at low concentration, the essential oil content in the olfactory bottle is 0.33–0.43 mg, and the gas mass concentration is 0.66–0.86 g/m^3^; at the medium concentration, the essential oil content in the olfactory bottle is 1.72–2.15 mg, and the gas mass concentration is 3.54–4.30 g/m^3^; at high concentration, the essential oil content in the olfactory bottle is 8.25–10.70 mg and the gas mass concentration is 16.50–21.40 g/m^3^. The ratio of essential oil content at low, medium, and high concentrations is about 1:5:25. An additional olfactory bottle without any odorant was added to the selection as a control.

### Chemical constituent analysis

The chemical constituents of essential oils were analyzed by gas chromatography–mass spectrometry (GC–MS, Agilent 7890B-5977A). A DB-WAX column (30 m × 0.25 mm × 0.25 μm) was utilized as a stationary phase. The GC conditions were as follows: helium was utilized as a mobile phase (1 ml/min); the splitting ratio was 30:1; the injector temperatures were held at 260°C; the oven temperature was programmed from 50°C to 120°C at 4°C/min, then held isothermally for 10 min and finally raised to 220°C at 2°C/min. The MS conditions were as follows: mass spectra were recorded with ionization energy of 70 eV and ion source temperature of 230°C.

The identification of the oil constituents was made by matching their recorded mass spectra with those stored in the NIST 14 mass spectral library of the GC–MS data system (Milman and Zhurkovich, 2016; Zhou et al., 2022).

### Questionnaire design

**Figure 1:**
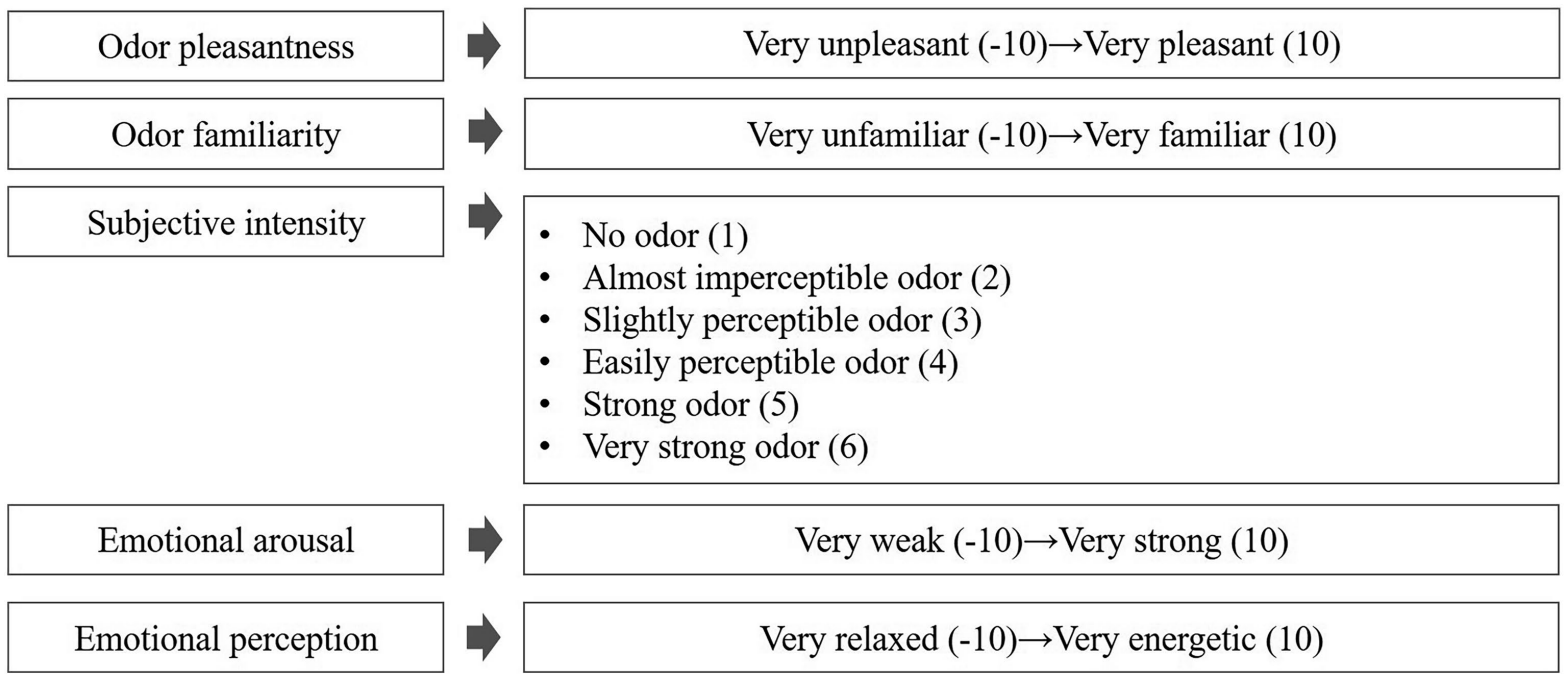
Contents of the questionnaire and specific evaluation index scores. The corresponding score range was shown in parentheses.

During the test, a questionnaire was used to investigate subjects ‘evaluation of odor, with five evaluation indicators of odor perception: pleasantness, familiarity, subjective intensity, emotional arousal and emotional perception, as shown in Figure 1. A pleasantness scale of −10 to 10 levels represents the pleasantness caused by odor from “very unpleasant” to “very pleasant.” A familiarity scale of −10 to 10 levels represents the pleasantness caused by odor from “very unfamiliar” to “very familiar.” For the subjective intensity, scores of 1–6, respectively, represent “no odor,” “almost imperceptible odor,” “slightly perceptible odor,” “easily perceptible odor,” “strong odor,” and “very strong odor.” An emotional perception scale of −10 to 10 levels represents the emotional perception caused by odor from “very relaxed” to “very energetic.” An arousal scale of −10 to 10 levels represents the arousal caused by odor from very weak to very strong. Among them, pleasantness and arousal are thought of as two independent dimensions of emotions (Russell, 1980; Feldman Barrett and Russell, 1998). Pleasantness reflects the pleasant-unpleasant properties of emotional stimuli, whereas emotional arousal reflects the degree of emotions evoked by the odor, and emotional perception reflects the attribute categories of emotions evoked by the odor.

### Experimental procedure

The experiments were performed with the subjects sitting position in an air-conditioned (temperature 23 ± 2°C) room. The test room was well ventilated, no perfume, smoking, or other factors affected the results.

Participants need to evaluate three groups of odors in order: low, medium, and high. Each odor group consisted of a total of 10 inhalation bottles including six essential oil odor samples, three blended essential oil odor samples, and one blank odor sample. The interval between each group trial was at least 10 min. To avoid the influence of olfactory sequence on odor evaluation, the olfactory sequences varied between the groups and the olfactory sequences would change again after every ten participants completed the experiment. Each odor sample was newly prepared for different subjects. Participants were instructed to remove the screw cap from each sample in turn, sniff odor 1 cm away from the opening of the bottle for 2–3 s, then immediately tighten the cap and fill in the questionnaire to do the odor evaluation. They could re-sniff the samples if they wished. The sniffing interval for each of the two odor samples was 60 s. When the participants finished evaluating a set of odor samples, they needed to leave the test room for 20 min for a rest, while the room was ventilated for 20 min.

### Statistical method

IBM SPSS Statistics 22.0 was used to establish a database with all results. All scale data are reported as means ± standard error of the mean (SEM). Two-factor repeated analyses of variance (ANOVAs) were run to analyze the effects of odor type and concentration on the sensory evaluation, and a *post hoc* Bonferroni test was used. At low, middle, and high concentrations, two-factor mixed analyses of variance (ANOVAs) were used to further analyze whether the correlation between odor sensory evaluation and type was influenced by gender, regional culture, or fragrance usage habits. Pearson’s correlation coefficients were used to assess relationships between five evaluation indexes of odor perception: pleasantness, familiarity, emotional perception, arousal, and subjective intensity.

## Results

### Participants’ characteristics

Basic information about participants’ age, hometown, and fragrance usage habits was collected and kept strictly confidential (Table 1). Based on the geographical location of the participants’ hometowns, the participants were divided into two categories: southern Yangtze (*n* = 25, hometowns located south of the Yangtze River in China) and northern Yangtze (*n* = 25, hometowns located north of the Yangtze River in China). In terms of fragrance usage habits, 24 participants selected “yes” to the question of whether they had fragrance usage habits, and they all use fragrance products (including essential oils, perfumes, scented candles, reed diffusers, etc.) more than 2–3 times a month. The 24 participants were divided into groups with fragrance usage habits, while the other 26 participants were divided into groups without fragrance usage habits.

**Table 1:** Basic information of participants.

Basic information		Number
Gender	Male	25
	Female	25
Hometowns	Southern Yangtze	25
	Northern Yangtze	25
Fragrance usage habits	Yes	24
	No	26

### Chemical constituents of essential oils

**Figure 2:**
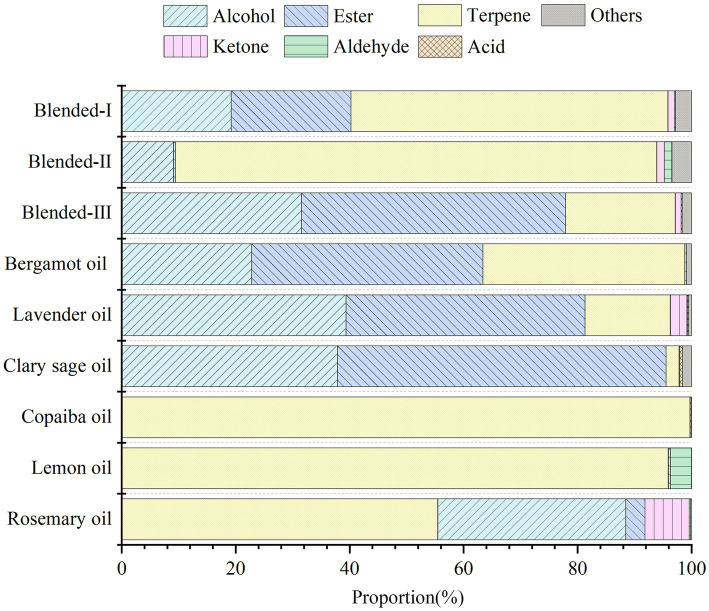
Distribution characteristics of compound types in different odors.

The analysis of the essential oil constituents was carried out by GC–MS with the peak area normalization method to clarify the relative content of each component (Figure 2). The main constituents of each essential oil were listed in Table 2 and a more detailed chemical constituent table can be found in Supplementary Tables S1–S9.

**Table 2:** The essential oil used in the study and their main constituents.

Essential oil	Chemical type	Main constituents
Lavender oil	Ester + Alcohol-type	Linalyl acetate (33.59%), Linalool (31.76%)
Clary sage oil	Ester + Alcohol-type	Linalyl acetate (50.46%), Linalool (26.20%), α-Terpineol (7.02%)
Bergamot oil	Ester + Alcohol-type	Linalyl acetate (40.55%), Limonene (33.31%), Linalool (20.88%)
Lemon oil	Terpene-type	Limonene (62.37%), β-Pinene (14.40%), γ-Terpinene (11.20%)
Rosemary oil	Terpene-type	α-Pinene (26.81%), Eucalyptol (25.99%), Camphene (7.88%), Camphor (6.11%)
Copaiba oil	Terpene-type	Caryophyllene (55.93%), α-Copaene (10.41%), trans-α-Bergamotene (6.81%), Humulene (5.459%)
Blended oil-I	Blended type	Linalyl acetate (19.33%), Limonene (17.39%), Caryophyllene (11.95%), Linalool (11.23%), Pinene (7.36%)
Blended oil-II	Terpene-type	Limonene (20.41%), Caryophyllene (19.67%), α-Pinene (12.24%), Eucalyptol (8.09%)
Blended oil-III	Ester + Alcohol-type	Linalyl acetate (41.43%), Linalool (25.81%), Limonene (12.10%)

Terpene-type, the main constituents of essential oil were terpene; Ester + Alcohol-type, the main constituents of essential oil were ester and alcohol. Blended type, the main constituents of essential oil were balanced. Main constituents: chemical constituents with a relative percentage over 5% in oil.

Nine essential oils could be classified into three types. One includes LVO, CSO, BGO, and blended-III, of which esters and alcohols were the main constituents. In LVO, esters accounted for 41.92%, alcohols 39.38%, and terpenes 14.99%. In CSO, esters accounted for 57.69%, alcohols 37.86%, and terpenes 2.28%. In BGO, esters accounted for 40.55%, alcohols 22.84%, and terpenes 35.45%. In blended-III, esters accounted for 31.52% and alcohols 46.4%. Linalool and linalyl acetate were the representative constituents of these essential oils. The second includes LMO, RMO, CPO, and blended-II, of which terpenes were the main constituents. The terpenes in LMO accounted for 95.78%, the highest content of which was limonene (62.37%); the terpenes in RMO accounted for 55.33%, the highest content of which was α-pinene (26.81%); the terpenes in CPO accounted for 99.60%, the highest content of which was caryophyllene (55.93%); and the main constituents in blended-II were also terpenes (84.07%), the higher content of which was limonene (20.41%) and caryophyllene (19.47%). The third group includes blended-I with a more average percentage of the three compounds, with 21.00% of esters, 19.23% of alcohols, and 55.65% of terpenoids.

### Effect of odor type and concentration on odor sensory evaluation

The main effect of odor types on odor sensory evaluation was significant (all *p*<0.05), while the main effect of concentration was not (all *p*>0.05). The interactive effects of odor types and intensity on pleasantness, familiarity, subjective intensity, emotional arousal, and emotional perception were not statistically significant (all *p*>0.05; Table 3).

**Table 3:** The significance of the indicators under main effect and interaction.

Source	Pleasantness	Familiarity	Subjective intensity	Emotional arousal	Emotional perception
Odor type	0.000*	0.000*	0.000*	0.000*	0.000*
Concentration	0.694	0.898	0.155	0.605	0.086
Odor type × Concentration	0.207	0.101	0.321	0.458	0.504

*represents significant difference (*p* < 0.05).

As the concentration increased, the mean scores of odor pleasantness and familiarity decreased, and the mean score of emotional perception increased, indicating that the perception of odors shifted to exciting energized. The mean score of arousal and subjective intensity also showed an increasing shift. However, those changes did not reach statistical significance, and none of the main effects of the concentration factors on odor sensory evaluation were statistically significant (Table 3). It was worth mentioning that the subjective intensity scores of the blank group were significantly lower than those of the experimental groups at all three concentrations (all *p* < 0.001). The detailed figure can be found in Supplementary Figure S1.

Average scores of odor sensory evaluation and further *post hoc* comparison results between the nine odors were shown in Table 4. In terms of odor pleasantness, the scores of different essential oil odors varied significantly [*F* (8, 392) = 18.407, *p* < 0.001]. LMO odor scored the highest, followed by odors of CPO, blended-II, BGO, RMO, blended-I, blended-III, and LVO. CSO odor scored lower than all other odors (all *p* < 0.05). Significant differences in familiarity were observed across odor samples [*F* (8, 392) =13.249, *p* < 0.001]. The familiarity score of LMO odor was significantly higher than those of other essential oils (all *p* < 0.001), while the familiarity score of LVO was the lowest.

**Table 4:** Sensory evaluation of different oil odor.

Essential oil odor	Pleasantness	Familiarity	Subjective intensity	Emotional arousal	Emotional perception
Blended-I	1.24 ± 0.43^c^	2.86 ± 0.55^b^	4.69 ± 0.10^ab^	2.79 ± 0.36^bc^	0.41 ± 0.42^bc^
Blended-II	2.01 ± 0.42^bc^	2.76 ± 0.47^b^	4.35 ± 0.10^c^	2.35 ± 0.36^c^	−0.17 ± 0.43^c^
Blended-III	1.15 ± 0.47^c^	2.54 ± 0.53^b^	4.72 ± 0.11^ab^	2.90 ± 0.35^bc^	0.56 ± 0.47^bc^
LVO	0.86 ± 0.44^cd^	1.71 ± 0.56^b^	4.75 ± 0.09^ab^	2.53 ± 0.45^bc^	0.29 ± 0.43^bc^
CSO	0.35 ± 0.50^d^	2.27 ± 0.54^bc^	4.80 ± 0.08^a^	3.07 ± 0.41^b^	0.79 ± 0.39^b^
BGO	1.84 ± 0.48^bc^	2.83 ± 0.51^b^	4.57 ± 0.10^b^	3.23 ± 0.34^b^	0.53 ± 0.45^b^
LMO	5.31 ± 0.43^a^	5.79 ± 0.46^a^	4.57 ± 0.10^b^	5.28 ± 0.37^a^	2.19 ± 0.54^a^
RMO	1.30 ± 0.47^c^	2.82 ± 0.55^b^	4.68 ± 0.11^ab^	3.20 ± 0.50^b^	1.03 ± 0.44^ab^
CPO	2.31 ± 0.39^b^	2.99 ± 0.55^b^	4.36 ± 0.09^c^	2.73 ± 0.37^bc^	−0.09 ± 0.42^c^

Mean ratings (±standard error of the mean, *n* = 50) of different odors at three concentrations on pleasantness, familiarity, subjective intensity, emotional arousal, and emotional perception. Values followed by different or same lowercase letters indicate significant differences (*p* < 0.05) or no significant difference (*p* > 0.05) between groups. Data were analyzed by main effects analysis with two-way repeated ANOVAs, a post hoc Bonferroni test was used.

The type of oil odor also influenced participants’ judgments of odor intensity [F (8, 392) =9.746, *p* < 0.001]. CPO and blended-II odors were perceived as the least concentrated odors (both *p*<0.05), while participants rated the highest odor intensity for the CSO odor. The emotional perception varied with the type of essential oil odor [*F* (8, 392) =2.490, *p* < 0.05]. Among them, LMO odor was rated as the most stimulating odor, only blended-II odor and CPO scored negatively, with their emotional perception being more inclined to calm and relax. In terms of arousal, LMO odor scored significantly higher than other oil odors (all *p* < 0.001), while blended-II odor scored lowest.

When the main constituent of the odors was considered instead of the specific type, the results were shown in Table 5. The analysis suggested that the terpene-type odors brought more energetic perception (*p* < 0.01) and higher emotional arousal (p < 0.001) than the other two odors. However, there was no significant difference in odor pleasantness, familiarity, and subjective intensity among the three.

**Table 5:** Sensory evaluation of different odor types.

Odor type	Pleasantness	Familiarity	Subjective intensity	Emotional arousal	Emotional perception
Blended type	1.95 ± 0.48^a^	2.86 ± 0.55^a^	4.69 ± 0.10^a^	2.79 ± 0.36^b^	0.41 ± 0.42^b^
Ester + Alcohol-type	1.30 ± 0.45^a^	2.54 ± 0.53^a^	4.75 ± 0.09^a^	2.52 ± 0.45^b^	0.29 ± 0.43^b^
Terpene-type	1.14 ± 0.40^a^	2.76 ± 0.47^a^	4.57 ± 0.10^a^	5.28 ± 0.37^a^	2.19 ± 0.54^a^

Mean ratings (± standard error of the mean, *n* = 50) of different odor types at three concentrations on pleasantness, familiarity, subjective intensity, emotional arousal, and emotional perception. Values followed by different or same lowercase letters indicate significant differences (*p* < 0.05) or no significant difference (*p* > 0.05) between groups. Data were analyzed by main effects analysis with two-way repeated ANOVAs, a post hoc Bonferroni test was used.

According to the Pearson correlation analysis results (Table 6), there was a positive correlation between familiarity and pleasantness (*r* = 0.531, *p* < 0.001). The higher the familiarity with the odor, the more popular it will be. In addition, there was a weaker positive correlation between emotional perception and subjective intensity (*r* = 0.328, *p* < 0.001). The analysis did not reveal any significant correlations between arousal and other subjective rating indicators.

**Table 6:** Correlation between sensory evaluation indexes.

PCCS	Pleasantness	Familiarity	Subjective intensity	Emotional arousal	Emotional perception
Pleasantness	1	0.531***	−0.267***	0.217***	−0.190***
Familiarity	0.531***	1	−0.007	0.290***	−0.900***
Subjective intensity	−0.267***	−0.007	1	0.211***	0.328***
Emotional arousal	0.217***	0.290***	0.211***	1	0.190***
Emotional perception	−0.190***	−0.090***	0.328***	0.190***	1

Pearson’s correlation coefficients (PCCS) were used to assess relationships between five evaluation indicators. ***p<0.001 represents that the confidence level of the results was more than 99.9%.

### Effect of participants’ characteristics on odor sensory evaluation

#### Influence of gender

**Figure 3:**
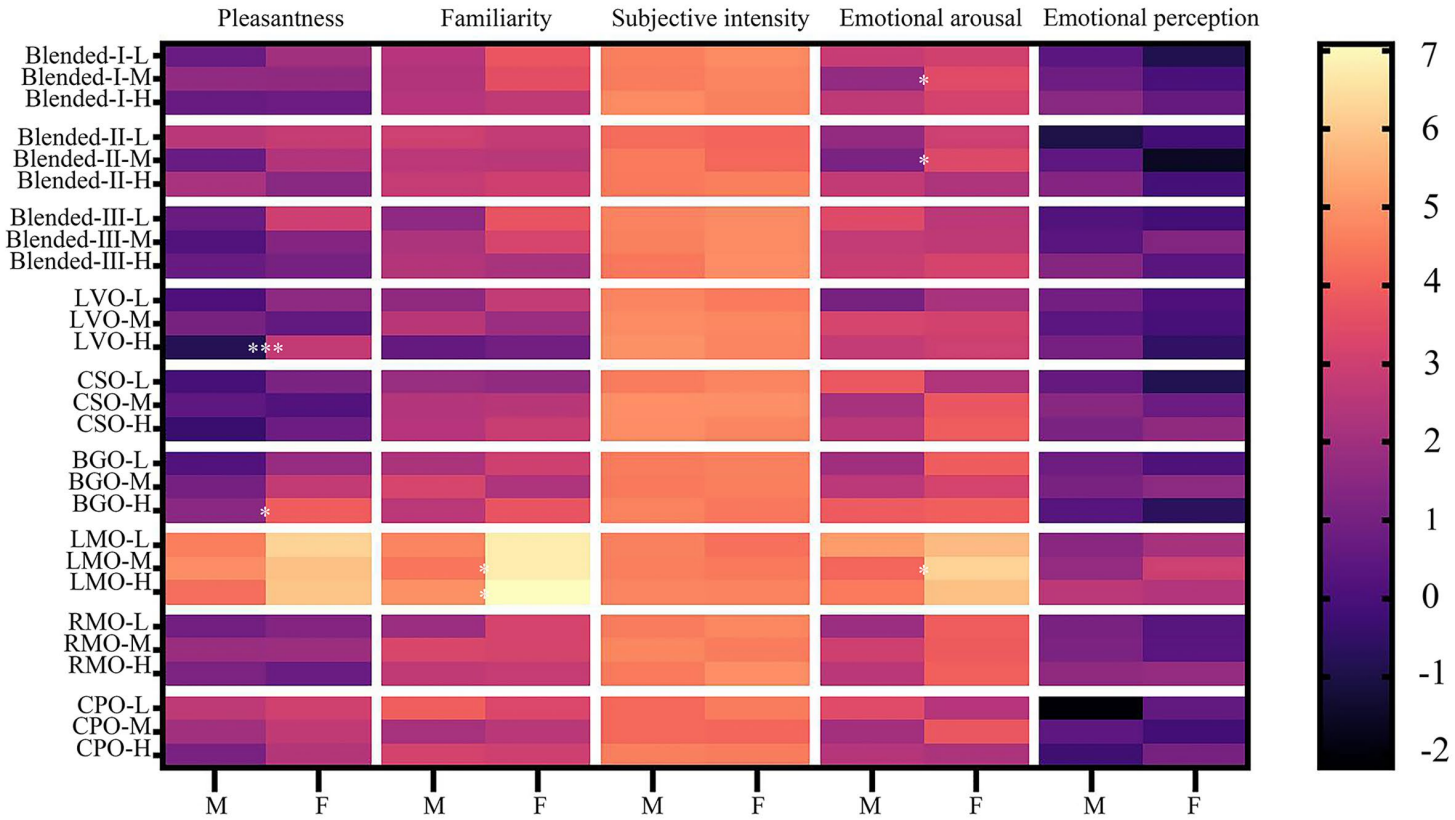
Sensory evaluation of odor between male and female. Mean ratings (*n* = 25) on pleasantness, familiarity, subjective intensity, emotional arousal, and emotional perception at low (L), mid-range (M), and high (H) concentrations between male and female. M: male, F: female. The asterisk indicates that the odor evaluation results of the two groups show significant differences. *p < 0.05, **p < 0.01, ***p < 0.001. Data were analyzed by main effects analysis with two-way mixed ANOVAs, a *post hoc* Bonferroni test was used.

The analysis results were shown in Table 7 and further *post hoc* comparison results were displayed in Figure 3. The interaction of gender and types had no significant effect on the odor sensory evaluation at three concentrations, respectively. At low concentrations, females scored higher than males on pleasantness for all odors [*F* (1, 48) =2.041, *p*>0.05] and the mean pleasantness scores of females were all positive at three concentrations while the male participants scored negatively for LVO and CSO odor at high concentrations. In addition, the females scored higher than males on average at three concentrations on odor familiarity [Low: *F* (1, 48) =0.697, Mid: *F* (1, 48) =0.173, High: *F* (1, 48) =0.286, all *p*>0.05] and arousal [Low: *F* (1, 48) =0.632, Mid: *F* (1, 48) =3.038, High: *F* (1, 48) =0.580, all *p*>0.05], and scored lower in emotional perception[*F* (1, 48) =0.036, Mid: *F* (1, 48) =0.158, High: *F* (1, 48) =0.398, all *p*>0.05], indicating that their emotional perception was more inclined to calm and relax. However, those differences did not reach statistical significance and the main effect of gender was not significant on odor sensory evaluation.

**Table 7:** The significance of the indicators under main effect and interaction of gender and type.

Odor concentration	Source	Pleasantness	Familiarity	Subjective intensity	Emotional arousal	Emotional perception
Low	Odor type	0.000*	0.000*	0.006*	0.000*	0.080*
	Gender	0.160	0.408	0.727	0.431	0.851
	Odor type × Gender	0.871	0.331	0.411	0.052	0.210
Middle	Odor type	0.000*	0.000*	0.000*	0.000*	0.024*
	Gender	0.412	0.680	0.852	0.088	0.692
	Odor type × Gender	0.655	0.221	0.739	0.193	0.476
High	Odor type	0.000*	0.000*	0.222	0.001*	0.014*
	Gender	0.095	0.595	1.000	0.450	0.531
	Odor type × Gender	0.074	0.582	0.055	0.612	0.487

Data represents the value of *p*, * significant difference at 0.05 level.

Further *post hoc* comparisons showed that at medium concentrations, the familiarity (6.76 ± 0.58 vs. 4.44 ± 0.88, *p* < 0.05) and arousal (6.24 ± 0.51 vs. 4.12 ± 0.68, *p* < 0.05) scores of LMO in female were significantly higher than those in male, and significant differences in arousal were also observed in blended-I (3.44 ± 0.58 vs. 1.68 ± 0.64, *p* < 0.05) and blended-II (3.36 ± 0.62 vs. 1.12 ± 0.75, *p* < 0.05). At high concentration, females scored significantly higher for pleasantness of LVO (2.71 ± 0.68 vs. −0.80 ± 0.72, *p* < 0.001) and BGO (3.92 ± 0.66 vs. 1.48 ± 0.72, *p* < 0.05) than males, and females were more familiar with LMO (7.08 ± 0.60 vs. 4.96 ± 0.60, *p* < 0.05).

#### Influence of regional culture

**Figure 4:**
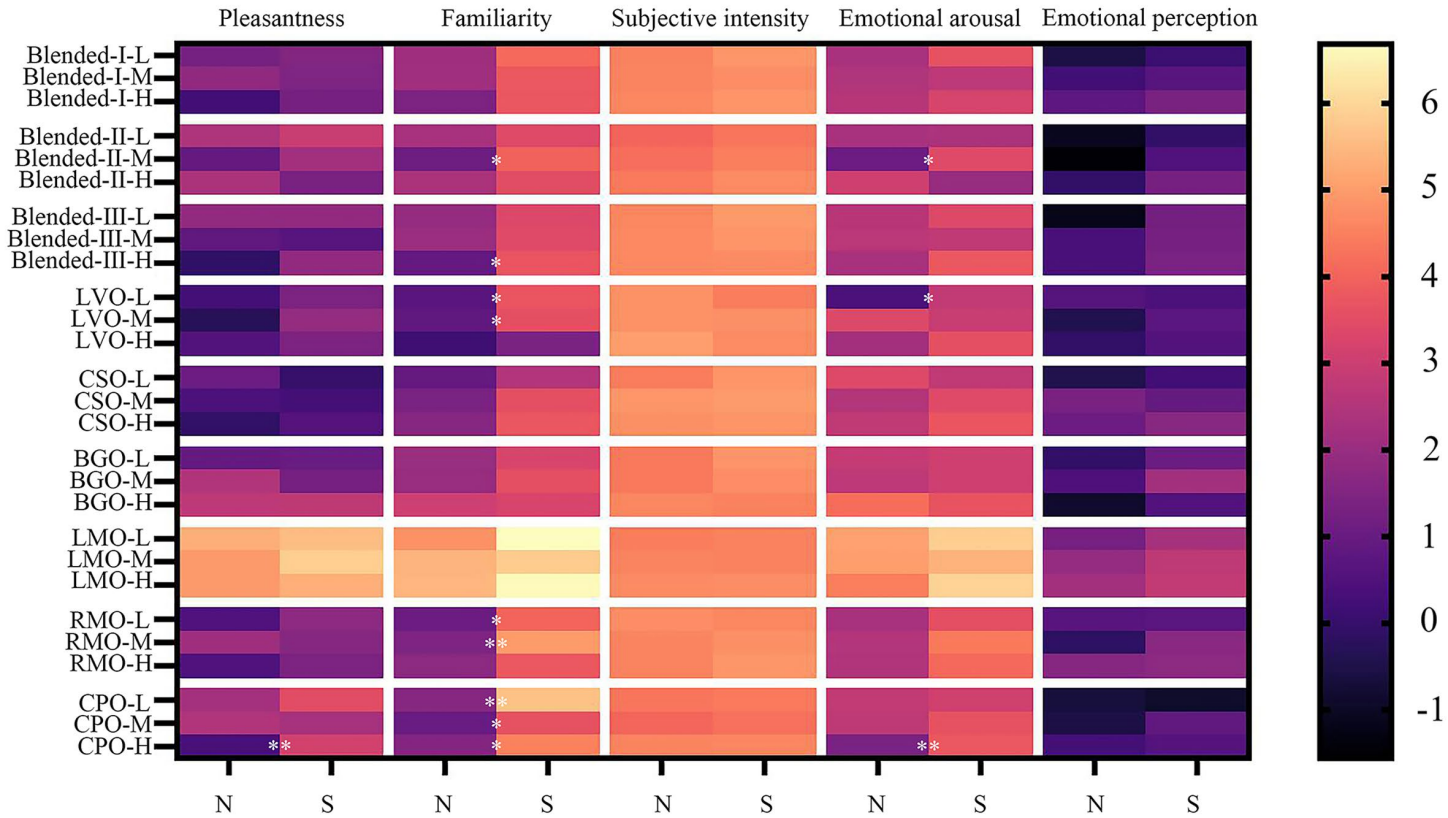
Effect of regional culture on sensory evaluation of odor. Mean ratings (*n* = 25) at three concentrations on pleasantness, familiarity, subjective intensity, emotional arousal, and emotional perception at low (L), mid-range (M), and high (H) concentration of the participants from the northern Yangtze and the southern Yangtze. N: participants from the northern Yangtze, S: participants from the southern Yangtze. The asterisk indicates that the odor evaluation results of the two groups show significant differences. * *p* < 0.05, ** *p* < 0.01. Data were analyzed by main effects analysis with two-way mixed ANOVAs, a *post hoc* Bonferroni test was used.

The analysis results were shown in Table 8 and the average ratings were obtained and further *post hoc* comparisons results were displayed in Figure 4. Similar to gender, the interaction between regional culture and type had no significant effect on odor sensory evaluation. The differences caused by regional culture were mainly reflected in odor familiarity. Participants’ ratings of odor familiarity varied significantly between regions at three concentrations [Low: *F* (1, 48) = 4.791, Mid: *F* (1, 48) =6.198, High: *F* (1, 48) = 4.613, all *p* < 0.05], with participants from the southern Yangtze giving higher rating (Low:1.92 ± 0.69 vs. 0.69 ± 0.30, Mid: 4.06 ± 0.59 vs. 4.00 ± 0.59, High: 3.79 ± 0.58 vs. 2.01 ± 0.59, all *p* < 0.05). Significant differences in familiarity were observed in LVO, RMO, CPO, blended-II, and blended-III, with the familiarity scores of CPO in participants from the southern Yangtze were all significantly higher than in participants from the northern Yangtze at three concentrations (Low:5.62 ± 0.50 vs. 1.60 ± 1.18, Mid: 3.60 ± 0.68 vs. 1.00 ± 1.00, High: 4.52 ± 0.50 vs. 1.56 ± 1.01, all *p* < 0.05).

**Table 8:** The significance of the indicators under main effect and interaction of region and type.

Odorconcentration	Source	Pleasantness	Familiarity	Subjective intensity	Emotional arousal	Emotional perception
Low	Odor type	0.019*	0.000*	0.006 *	0.000*	0.058
	Region	0.615	0.034*	0.292	0.186	0.315
	Odor type × Region	0.407	0.338	0.170	0.543	0.835
Middle	Odor type	0.000*	0.000*	0.000*	0.000*	0.025*
	Region	0.791	0.016*	0.350	0.262	0.165
	Odor type × Region	0.377	0.278	0.941	0.209	0.780
High	Odor type	0.000*	0.000*	0.322	0.000*	0.015*
	Region	0.176	0.037*	0.682	0.152	0.312
	Odor type × Region	0.321	0.314	0.411	0.064	0.98

Data represents the value of *p*, * significant difference at 0.05 level.

In terms of odor pleasantness, subjective intensity, emotional perception, and arousal, the average scores at three concentrations of participants from the southern Yangtze were all higher than those from the northern Yangtze. The significant differences in arousal between the two groups were observed in LVO (2.80 ± 0.78 vs. 0.40 ± 0.78, *p* < 0.05), blended-II (3.40 ± 0.65 vs. 1.08 ± 0.72, *p* < 0.05), and CPO (3.76 ± 0.59 vs. 0.92 ± 0.84, *p* < 0.01) at low, medium, and high concentrations, respectively. However, the main effects of these factors were not significant on odor sensory evaluation.

#### Influence of fragrance usage habits

**Figure 5:**
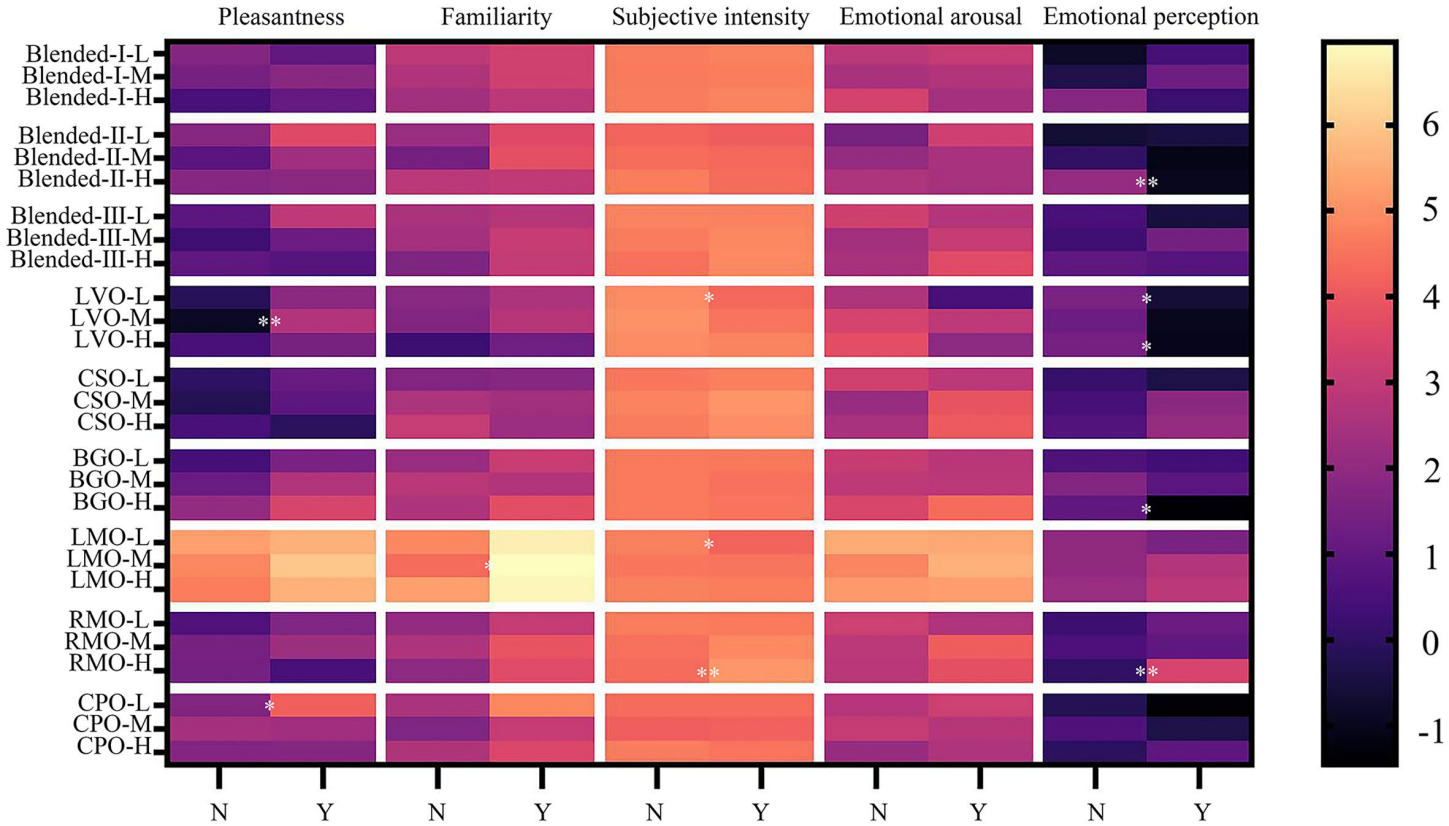
Effect of fragrance usage habits on sensory evaluation of odor. Mean ratings (*n* = 24–26) at three concentrations on pleasantness, familiarity, subjective intensity, emotional arousal, emotional perception and at low (L), mid-range (M), and high (H) concentration of the participants without fragrance usage habits and the participants with fragrance usage habits. N: participants without fragrance usage habits, Y: participants with fragrance usage habits. The asterisk indicates that the odor evaluation results of the two groups show significant differences. *p < 0.05, **p < 0.01. Data were analyzed by two-way mixed ANOVAs, a *post hoc* Bonferroni test was used.

The influence of fragrance usage habits on odor sensory evaluation was shown in Table 9 and the average ratings were obtained and further *post hoc* comparisons results were displayed in Figure 5. The main effects of fragrance usage habits on odor sensory evaluation were not significant at low and middle concentrations. At high concentrations, the interaction of fragrance usage habits and type had a significant effect on emotional perception [*F* (8, 384) =5.335, *p*<0.001] and subjective intensity [*F* (8, 384) =3.277, *p*<0.01].

**Table 9:** The significances of the indicators under main effect and interaction of habits and type.

Odorconcentration	Source	Pleasantness	Familiarity	Subjective intensity	Emotional arousal	Emotional perception
Low	Odor type	0.000*	0.000*	0.015*	0.000*	0.083
	Fragrance usage habits	0.136	0.325	0.434	0.732	0.623
	Odor type × Fragrance usage habits	0.419	0.686	0.229	0.302	0.497
Middle	Odor type	0.000*	0.000*	0.000*	0.000*	0.018*
	Fragrance usage habits	0.101	0.221	0.924	0.506	0.991
	Odor type × Fragrance usage habits	0.386	0.245	0.120	0.497	0.127
High	Odor type	0.000*	0.000*	0.250	0.000*	0.002*
	Fragrance usage habits	0.701	0.339	0.569	0.714	0.610
	Odor type × Fragrance usage habits	0.747	0.453	0.001 *	0.126	0.000*

Data represents the value of *p*, * significant difference at 0.05 level.

Further simple effects analysis revealed that participants with fragrance usage habits believed that the emotional perception caused by LVO odor, BGO odor, and blended-II odor was more inclined to calm and relax (all *p* < 0.05), while those without fragrance usage habits were the opposite. But the contrary difference was shown between the two groups for RMO odor, participants with fragrance usage habits perceived that the emotional perception was more inclined to encourage (2.83 ± 0.92 vs. 0.00 ± 0.75, *p* < 0.01). In terms of subjective intensity, participants with fragrance usage habits scored significantly higher for RMO odor (5.13 ± 0.19 vs. 4.35 ± 0.18, *p* < 0.01). In terms of odor pleasantness and familiarity, the average scores at three concentrations of participants with fragrance usage habits were all higher than participants without fragrance usage habits but those differences did not reach statistical significance.

The results of the correlation analysis showed that there was no significant correlation between the three factors of gender, region, and fragrance use habits, and a more detailed table can be found in Supplementary Table S10.

## Discussion

In this study, according to the constituent and function, nine essential oil odors with health function were selected for the sensory evaluation experiment at low, medium, and high concentrations, respectively. The evaluation indexes included pleasantness, familiarity, emotional perception, arousal, and subjective intensity. The effects of odor types, concentration, and their interactions were explored. Gender, hometown (regional culture), and fragrance habits factors were chosen to better understand how participants’ characteristics might impact the responses to odors.

The results showed that the odor type significantly affected the evaluation results, while concentration did not, and there was no interaction between the two factors. Essential oils are composed of various small molecular volatile chemical components, which also differ in sensory evaluation. A study on the odorant hedonic value of 23 monomeric compounds found that isoamyl acetate and geraniol had higher preference and familiarity scores than limonene, while limonene had a relatively low score of subjective intensity among all compounds (Chalencon et al., 2022). The results of the present study suggested that the terpene-type odors brought more inspiring perception and higher emotional arousal than the other two odors. The finding is consistent with prior literature that shows an effect of emotional perception triggered by chemical composition differences (Kaneda et al., 2011). One study found that strawberry odor had a relaxing effect, while lemon odor had a stimulating effect. This difference may also be related to the chemical composition, since the compositions of strawberry odor are mainly esters and alcohols while that of lemon odor is dominated by terpenes (Baccarani et al., 2021b). However, LMO, CPO, and blended-II were all terpene-type odors. LMO odor received the highest ratings for emotional perception and arousal, while CPO odor and blended-II odor received the lowest ratings with negative values, which showed a more calming and relaxing perception. It indicates that oil odors with similar major constituents may differ significantly in their taste, ingredient type was not the primary predictor of odor sensory evaluation.

In this experiment, odor concentration did not significantly affect the odor sensory evaluation. While the concentration increased, the participants’ subjective intensity remained between the levels of “easily perceptible odor” and “strong odor.” The subjective intensity scores of the blank group were significantly lower than those of the experimental groups at all three concentrations, indicating that the participants without olfactory training were able to correctly discriminate between the presence or absence of odors, but not well enough to discriminate the changes in odor concentration. It was noteworthy that there were significant differences in subjective intensities between the different odors, with the highest subjective intensity score for CSO odor and the lowest subjective intensity score for the CPO odor and blended II odor, which may be related to the nasal pungency of the different odors. One previous study showed that pungency contributed to overall aroma intensity (Jin et al., 2018), which was also reflected by the highest subjective intensity score for CSO odor in this experiment. However, in another study, the subjective intensity scores of different odors were very close (Ba and Kang, 2019). This inconsistency of results might be caused by different odor types and experimental tasks design.

People’s characteristics influenced odor sensory evaluation to some extent. Gender differences in the ability to detect, discriminate, and identify odors are still a matter of debate. Previous studies have shown that females possess higher olfactory sensitivity than males and there were significant gender differences for the hedonic estimate (Thuerauf et al., 2009; Greenberg et al., 2013). However, in the present study, significant differences were observed only in some oil odor such as LMO, the main effect of gender was not significant on odor sensory evaluation, which is in agreement with past studies (Van Thriel et al., 2008; Nováková et al., 2014), and there was no interaction between the gender and type.

China’s Yangtze River is known as the mother river of the Chinese nation, which flows from west to east to debouch into the East China Sea. It has served as an important link between nature and people. The results of this study showed that the differences in odor sensory evaluation between participants from the southern Yangtze and the northern Yangtze were mainly reflected in familiarity. At three concentrations, participants from the southern Yangtze were more familiar with the odor than participants from the northern Yangtze. These differences presumably reflected vegetation differences caused by climates. The climate in the region south of the Yangtze River is dominated by a subtropical monsoon climate with ample light and heat resources, which is suitable for the growth of lavender, rosemary, bergamot, and lemon. Both lemon and bergamot are cold-intolerant and rarely cultivated in the northern Yangtze, which is presumably related to the difference between the two groups in odor sensory evaluation.

Fragrance usage habits also influenced people’s odor evaluation. People with fragrance usage habits showed a higher level of pleasantness and familiarity with essential oil odors than those without fragrance usage habits, but those differences did not reach statistical significance. This may be related to the difference in odor between essential oils and perfumes. Previous studies reported that exposure to relatively high concentrations of chemicals affected sensitivity to that particular odorant (Zibrowski and Robertson, 2006; Sorokowska et al., 2013), but our study showed that the fragrance usage habits did not significantly affect subjective intensity. It may be related to the concentration difference. In daily life, people perceive a relatively lower concentration of fragrance indoors compared to the chemicals. In addition, at high concentrations, participants with fragrance usage habits perceived the emotional perception associated with LVO odor, BGO odor, and blended-II odor as more calming and relaxing, while the perception associated with RMO odor was more motivating and inspiring, but those without fragrance usage habit were the opposite. Those differences may be related to the prior experience of participants with fragrance usage habits. Once participants identified the type of odor, their emotional perception of the odor may have been potentially influenced by pre-existing understanding. The findings in this study are helpful to the field of aromatherapy for more personalized treatments can be performed. In addition to considering the possible efficacy of essential oil odor, factors such as chemical type, gender, hometown, and fragrance usage habits should be taken into comprehensive consideration in the selection of oil odor, which may improve people’s experience in aromatherapy and better help people improve their mood.

Subjective odor perception is often investigated with the help of several dimensions, such as pleasantness, familiarity, intensity, emotional perception, and arousal. Studies have demonstrated that these dimensions are not independent, especially between familiarity and pleasantness. Studies have generally found that the more familiar an odor, the more pleasant it is judged (Knaapila et al., 2017). However, it had also been suggested that the relation between pleasantness and familiarity was specific to pleasant odors (Delplanque et al. 2008). In this study, the mean pleasantness scores for all odors were positive and those odors could be considered not to be unpleasant odors. And the correlation analysis revealed that there was a positive correlation between familiarity and pleasantness, which agrees with the univariate analyses. In addition, there was a weaker positive correlation between emotional perception and subjective intensity, implying that the odor properties of inspiring were associated with higher subjective intensity, in line with the previous findings (Baccarani et al., 2021a).

However, this study has potential limitations. Many studies have shown that age affects odor perception. In this experiment, the odor sensory evaluation test was conducted for young adults, so it is unclear whether age will affect the oil odor evaluation results. Age factor can be added as a variable in future research. On the other hand, the odor sensory evaluations were obtained based on participants’ short-term sniffing in this experiment. Although participants were allowed to sniff repeatedly if they wished, it is still unclear whether the evaluation changed after long-term sniffing. A comparison between short-term sensory evaluation and long-term sensory evaluation could be discussed in future studies. Meanwhile, the sample size of this study was limited, which may affect the statistical significance of the results.

## Conclusion

Essential oils are widely used as functional fragrances to improve people’s olfactory environment and regulate emotions. In this study, nine oil odors were selected based on chemical composition and function. The effects of odor conditions, participants’ characteristics on odor sensory evaluation, and the interactions between different evaluation indexes were investigated.

Chemical type, but not the concentration, significantly influenced the evaluation. There was no interaction between the two factors. The terpene-type odors brought more inspiring perception and higher emotional arousal than the ester+alcohol-type odors and the blended odors, but significant differences also existed between odors with the same main constituent, indicating that constituent type was not the primary predictor of odor sensory evaluation.

Significant geographical differences for odor familiarity existed, with participants from the southern Yangtze scoring significantly higher than participants from the northern Yangtze. Fragrance usage habits significantly influenced the subjective intensity and emotional perception ratings of some odors. There was no significant gender difference in odor sensory evaluation; In addition, familiarity and pleasantness were positively correlated, and emotional perception and subjective intensity also showed a weak correlation.

## Data availability statement

The original contributions presented in the study are included in the article/Supplementary material, further inquiries can be directed to the corresponding authors.

## Ethics statement

The studies involving human participants were reviewed and approved by the Research and Ethics Offices of the Shanghai Jiao Tong University. The patients/participants provided their written informed consent to participate in this study.

## Author contributions

JC: methodology, investigation, project administration, formal analysis, and writing original draft. NZ: investigation, formal analysis, project administration, supervision, and funding acquisition. SP: investigation and formal analysis. LY: conceptualization, supervision and funding acquisition. All authors contributed to the article and approved the submitted version.

## Funding

This work was supported by the National Key Research and Development Program of China (grant number: 2019YFA0706200), Shanghai Sailing Program (grant number: 20YF1421700), and National Natural Science Foundation of China (grant number: 52078291).

## Conflict of interest

The authors declare that the research was conducted in the absence of any commercial or financial relationships that could be construed as a potential conflict of interest.

## Publisher’s note

All claims expressed in this article are solely those of the authors and do not necessarily represent those of their affiliated organizations, or those of the publisher, the editors and the reviewers. Any product that may be evaluated in this article, or claim that may be made by its manufacturer, is not guaranteed or endorsed by the publisher.
