# Supplementary material for: Odor perception of aromatherapy essential oils with different chemical types: Influence of gender and two cultural characteristics
Source: Front Psychol. 2022 Nov 10;13:998612. doi: 10.3389/fpsyg.2022.998612 (PMC9686375; doi:10.3389/fpsyg.2022.998612)
Supplement: Supplementary file 1 [file Table_1.DOCX]

Supplementary Material

# Supplementary Tables and Figures

## Supplementary Tables

# A. Main chemical compositions and relative content of essential oil

# Supplementary Table 1 Main chemical compositions and relative content of lavender oil

| NO | Chemical composition | Retention time | Peak area% |
| --- | --- | --- | --- |
| 1 | Linalyl acetate | 19.90 | 33.59 |
| 2 | Linalool | 19.72 | 31.76 |
| 3 | β-Caryophyllene | 20.90 | 4.39 |
| 4 | β-Famesene | 23.46 | 3.48 |
| 5 | Lavandulyl acetate | 21.38 | 3.31 |
| 6 | 3-Octanone | 10.36 | 2.44 |
| 7 | Ocimene | 10.27 | 2.14 |
| 8 | trans-β-Ocimene | 9.76 | 2.12 |
| 9 | Terpinen-4-ol | 21.21 | 1.79 |
| 10 | 1-Octen-3-yl-acetate | 14.36 | 1.28 |
| 11 | α-Terpineol | 24.84 | 1.27 |
| 12 | Hexyl acetate | 10.96 | 1.11 |
| 13 | Lavandulol | 24.06 | 1.08 |
| 14 | Borneol | 24.97 | 1.05 |
| 15 | Eucalyptol | 8.92 | 0.77 |
| 16 | Myrcene | 7.63 | 0.70 |
| 17 | Hexyl butyrate | 15.52 | 0.57 |
| 18 | 3-Octanol, acetate | 13.01 | 0.53 |
| 19 | Geranyl acetate | 27.86 | 0.52 |
| 20 | 3-Octanol | 14.95 | 0.50 |
| 21 | α-Santalene | 20.24 | 0.49 |
| 22 | Limonene | 8.59 | 0.38 |
| 23 | Neryl acetate | 26.18 | 0.38 |
| 24 | β-Phellandrene | 8.85 | 0.37 |
| 25 | Geraniol | 34.19 | 0.33 |
| 26 | Linalool oxide | 16.36 | 0.32 |
| 27 | Cryptone | 23.32 | 0.29 |
| 28 | Caryophyllene oxide | 40.56 | 0.21 |
| 29 | Hexyl butyrate | 9.28 | 0.19 |
| 30 | 1-Octen-3-ol | 16.70 | 0.18 |
| 31 | 1-Hexanol | 13.64 | 0.18 |
| 32 | trans-α-Bergamotene | 20.62 | 0.18 |
| 33 | Hexyl hexanoate | 21.50 | 0.17 |
| 34 | p-Cymene | 10.77 | 0.16 |
| 35 | Camphor | 18.39 | 0.16 |
| 36 | Nerol | 30.90 | 0.14 |
| 37 | Camphene | 5.12 | 0.12 |
| 38 | Bornyl acetate | 20.49 | 0.12 |
| 39 | α-Pinene | 4.27 | 0.12 |
| 40 | 3-Carene | 7.17 | 0.11 |
| Total | | 98.99 | |

# Note: The table shows the main compounds whose relative content was above 0.1%

# Supplementary Table 2 Main chemical compositions and relative content of clary sage oil

| NO | Chemical composition | Retention time | Peak area% |
| --- | --- | --- | --- |
| 1 | Linalyl acetate | 19.92 | 50.46 |
| 2 | Linalool | 19.73 | 26.20 |
| 3 | α-Terpineol | 24.86 | 7.02 |
| 4 | Geranyl acetate | 27.88 | 4.47 |
| 5 | Neryl acetate | 26.20 | 2.69 |
| 6 | Geraniol | 34.20 | 2.57 |
| 7 | Nerol | 30.91 | 1.25 |
| 8 | β-Caryophyllene | 20.90 | 0.79 |
| 9 | Caryophyllene oxide | 40.57 | 0.50 |
| 10 | Linalool oxide | 16.36 | 0.43 |
| 11 | α-Copaene | 17.73 | 0.42 |
| 12 | Myrcene | 7.63 | 0.40 |
| 13 | Limonene | 8.59 | 0.26 |
| 14 | β--Bourbonene | 18.54 | 0.19 |
| 15 | 3-Hexen-1-ol | 14.60 | 0.16 |
| 16 | β-Citral | 23.92 | 0.11 |
| Total | | 97.93 | |

# Note: The table shows the main compounds whose relative content was above 0.1%

# Supplementary Table 3 Main chemical compositions and relative content of bergamot oil

| NO | Chemical composition | Retention time | Peak area% |
| --- | --- | --- | --- |
| 1 | Linalyl acetate | 19.91 | 40.55 |
| 2 | Limonene | 8.64 | 33.31 |
| 3 | Linalool | 19.67 | 20.88 |
| 4 | α-Terpineol | 24.78 | 1.27 |
| 5 | p-Cymene | 10.76 | 0.77 |
| 6 | Myrcene | 7.62 | 0.46 |
| 7 | Plinol C | 20.66 | 0.38 |
| 8 | Linalool oxide | 16.32 | 0.26 |
| 9 | Citral | 26.18 | 0.25 |
| 10 | α-Pinene | 4.25 | 0.23 |
| 11 | β-Pinene | 6.07 | 0.19 |
| 12 | Sabinene | 6.42 | 0.16 |
| Total | | 98.71 | |

# Note: The table shows the main compounds whose relative content was above 0.1%

# Supplementary Table 4 Main chemical compositions and relative content of lemon oil

| NO | Chemical composition | Retention time | Peak area % |
| --- | --- | --- | --- |
| 1 | Limonene | 8.67 | 62.37 |
| 2 | β-Pinene | 6.08 | 14.41 |
| 3 | γ-Terpinene | 10.04 | 11.20 |
| 4 | α-Pinene | 4.25 | 2.61 |
| 5 | Citral | 26.19 | 2.46 |
| 6 | Sabinene | 6.42 | 2.24 |
| 7 | Myrcene | 7.62 | 1.45 |
| 8 | β-Citral | 23.87 | 1.11 |
| 9 | p-Cymene | 10.76 | 0.60 |
| 10 | Geranyl acetate | 27.83 | 0.34 |
| 11 | β-Phellandrene | 8.86 | 0.28 |
| 12 | Linalool | 19.62 | 0.12 |
| 13 | 3-Thujene | 4.33 | 0.12 |
| 14 | Terpinolene | 11.18 | 0.11 |
| 15 | 4-Carene | 8.02 | 0.10 |
| Total | | 99.52 | |

# Note: The table shows the main compounds whose relative content was above 0.1%

# Supplementary Table 5 Main chemical compositions and relative content of rosemary oil

| NO | Chemical composition | Retention time | Peak area % |
| --- | --- | --- | --- |
| 1 | α-Pinene | 7.97 | 26.82 |
| 2 | Eucalyptol | 11.54 | 25.99 |
| 3 | Camphene | 8.44 | 7.88 |
| 4 | Camphor | 15.85 | 6.11 |
| 5 | α-Phellandrene | 10.49 | 3.86 |
| 6 | β-Pinene | 9.44 | 3.78 |
| 7 | Bornyl acetate | 21.18 | 3.36 |
| 8 | Borneol | 16.68 | 2.98 |
| 9 | γ-Terpinene | 12.59 | 2.18 |
| 10 | β-Caryophyllene | 25.86 | 1.77 |
| 11 | Humulene | 26.99 | 1.67 |
| 12 | Verbenone | 18.31 | 1.67 |
| 13 | α-Terpineol | 17.63 | 1.64 |
| 14 | Myrcene | 10.03 | 1.60 |
| 15 | 4-Carene | 10.95 | 1.57 |
| 16 | Terpinolene | 13.72 | 1.31 |
| 17 | 3-Thujene | 7.71 | 1.27 |
| 18 | Terpinen-4-ol | 17.12 | 1.22 |
| 19 | p-Cymene | 11.26 | 0.83 |
| 20 | Linalool | 14.17 | 0.48 |
| 21 | Tricyclene | 7.51 | 0.32 |
| 22 | Thuja-2,4(10)-diene | 8.63 | 0.24 |
| 23 | Citronellol | 19.04 | 0.18 |
| 24 | Geraniol | 19.99 | 0.17 |
| 25 | 4-Thujanol | 12.87 | 0.14 |
| 26 | Sabinene | 9.35 | 0.14 |
| 27 | Myrtenol | 17.84 | 0.12 |
| Total | | 99.28 | |

# Note: The table shows the main compounds whose relative content was above 0.1%

# Supplementary Table 6 Main chemical compositions and relative content of copaiba oil

| NO | Chemical composition | Retention time | Peak area % |
| --- | --- | --- | --- |
| 1 | Caryophyllene | 20.07 | 55.93 |
| 2 | α-Copaene | 16.83 | 10.41 |
| 3 | trans-α-Bergamotene | 19.83 | 6.81 |
| 4 | Humulene | 22.27 | 5.46 |
| 5 | Germacrene D | 23.81 | 4.38 |
| 6 | δ-Cadinene | 26.06 | 2.00 |
| 7 | β-Bisabolene | 24.82 | 1.98 |
| 8 | α-Cubebene | 15.78 | 1.84 |
| 9 | 1,5-Cyclodecadiene | 21.34 | 1.55 |
| 10 | δ-Elemene | 16.15 | 1.34 |
| 11 | α-amorphene | 23.07 | 1.33 |
| 12 | Cadinene | 18.28 | 0.86 |
| 13 | β-Selinene | 24.16 | 0.74 |
| 14 | Cyperene | 17.77 | 0.49 |
| 15 | β-ylangene | 19.30 | 0.45 |
| 16 | α-Muurolene | 24.57 | 0.40 |
| 17 | Alloaromadendrene | 21.47 | 0.31 |
| 18 | β-Famesene | 22.96 | 0.57 |
| 19 | γ-Cadinene | 29.64 | 0.26 |
| 20 | α-ylangene | 16.52 | 0.22 |
| 21 | trans-β-Ocimene | 8.52 | 0.20 |
| 22 | α-Bisabolene | 27.19 | 0.16 |
| 23 | β-sesquiphellandrene | 26.84 | 0.13 |
| 24 | α-Gurjunene | 20.32 | 0.12 |
| 25 | Elemene isomer | 16.45 | 0.11 |
| 26 | Benzene | 26.94 | 0.11 |
| Total | | 98.14 | |

# Note: The table shows the main compounds whose relative content was above 0.1%

# Supplementary Table 7 Main chemical compositions and relative content of blended-Ⅰ

| NO | Chemical composition | Retention time | Peak area % |
| --- | --- | --- | --- |
| 1 | Linalyl acetate | 19.82 | 19.33 |
| 2 | Limonene | 8.58 | 17.39 |
| 3 | Caryophyllene | 20.88 | 11.95 |
| 4 | Linalool | 19.62 | 11.23 |
| 5 | Pinene | 4.24 | 7.36 |
| 6 | Eucalyptol | 8.88 | 4.77 |
| 7 | β-Pinene | 6.04 | 2.44 |
| 8 | Camphene | 5.07 | 1.88 |
| 9 | γ-Terpinene | 9.99 | 1.81 |
| 10 | Copaene | 17.71 | 1.72 |
| 11 | α-Limonene | 20.59 | 1.65 |
| 12 | Humulene | 23.26 | 1.64 |
| 13 | β-copaene | 24.89 | 1.43 |
| 14 | o-Cymene | 10.72 | 1.42 |
| 15 | α-Terpineol | 24.71 | 1.29 |
| 16 | Geraniol | 33.97 | 1.28 |
| 17 | Camphor | 18.31 | 0.78 |
| 18 | δ-Cadinene | 27.41 | 0.77 |
| 19 | β-Famesene | 23.40 | 0.73 |
| 20 | β-Bisabolene | 25.96 | 0.66 |
| 21 | Geranyl acetate | 27.72 | 0.61 |
| 22 | Lavandulyl acetate | 21.30 | 0.43 |
| 23 | γ-Muurolene | 24.12 | 0.42 |
| 24 | γ-Elemene | 22.24 | 0.41 |
| 25 | β-Elemene | 20.69 | 0.41 |
| 26 | sabinene | 6.39 | 0.37 |
| 27 | α-Cubebene | 16.69 | 0.37 |
| 28 | Terpinen-4-ol | 21.12 | 0.36 |
| 29 | β-Ocimene | 10.22 | 0.33 |
| 30 | 3-Octanone | 10.29 | 0.32 |
| 31 | trans-β-Ocimene | 9.71 | 0.30 |
| 32 | Caryophyllene oxide | 40.41 | 0.30 |
| 33 | δ-Elemene | 17.07 | 0.25 |
| 34 | β-cyclocitral | 23.81 | 0.20 |
| 35 | Terpinene | 7.99 | 0.18 |
| 36 | γ-Muurolene | 20.19 | 0.17 |
| 37 | 1-Octen-3-yl-acetate | 14.30 | 0.16 |
| 38 | Nerol | 30.68 | 0.15 |
| 39 | Bornyl acetate | 20.41 | 0.15 |
| 40 | β-selinene | 25.31 | 0.15 |
| 41 | Acetic acid, hexyl ester | 10.90 | 0.14 |
| 42 | Alloaromadendrene | 22.42 | 0.14 |
| 43 | Terpinolene | 11.14 | 0.11 |
| Total | | 97.95 | |

# Note: The table shows the main compounds whose relative content was above 0.1%

# Supplementary Table 8 Main chemical compositions and relative content of blended-Ⅱ

| NO | Chemical composition | Retention time | Peak area % |
| --- | --- | --- | --- |
| 1 | Limonene | 8.60 | 20.41 |
| 2 | Caryophyllene | 20.89 | 19.67 |
| 3 | α-pinene | 4.25 | 12.24 |
| 4 | Eucalyptol | 8.90 | 8.09 |
| 5 | β-Pinene | 6.04 | 4.16 |
| 6 | Camphene | 5.08 | 3.29 |
| 7 | α-Copaene | 17.72 | 3.08 |
| 8 | γ-Terpinene | 9.99 | 3.03 |
| 9 | Humulene | 23.27 | 2.96 |
| 10 | trans-α-Bergamotene | 20.60 | 2.85 |
| 11 | o-Cymene | 10.72 | 2.28 |
| 12 | Germacrene D | 24.90 | 2.12 |
| 13 | δ-Cubebene | 27.41 | 1.41 |
| 14 | β-Bisabolene | 25.97 | 1.35 |
| 15 | Bornanone | 18.31 | 1.31 |
| 16 | Sabinene | 7.59 | 1.02 |
| 17 | Citral | 26.09 | 0.98 |
| 18 | γ-Muurolene | 24.12 | 0.80 |
| 19 | γ-Elemene | 22.24 | 0.77 |
| 20 | β-elemene | 20.70 | 0.70 |
| 21 | α-Cubebene | 16.69 | 0.64 |
| 22 | Sabinene | 6.39 | 0.60 |
| 23 | Verbenone | 24.70 | 0.43 |
| 24 | Caryophyllene oxide | 40.41 | 0.42 |
| 25 | β-Citral | 23.81 | 0.34 |
| 26 | Terpinene | 7.99 | 0.30 |
| 27 | Alloaromadendrene | 25.31 | 0.30 |
| 28 | Alloaromadendrene | 22.42 | 0.26 |
| 29 | α-Muurolene | 25.74 | 0.23 |
| 30 | Terpinen-4-ol | 21.12 | 0.21 |
| 31 | Bornyl acetate | 20.39 | 0.21 |
| 32 | Junenol | 44.73 | 0.19 |
| 33 | Cyperene | 18.72 | 0.17 |
| 34 | Clovene | 20.16 | 0.17 |
| 35 | α-Selinene | 25.56 | 0.14 |
| 36 | Baicalene | 23.06 | 0.14 |
| 37 | α-bisabolene | 28.54 | 0.14 |
| 38 | Linalool | 19.58 | 0.13 |
| 39 | γ-Elemene | 31.36 | 0.13 |
| 40 | Terpinolene | 11.14 | 0.13 |
| 41 | Geranyl acetate | 27.72 | 0.12 |
| 42 | 3-Carene | 3.99 | 0.12 |
| 43 | α-curcumene | 28.38 | 0.11 |
| 44 | Ylangene | 17.43 | 0.11 |
| Total | | 98.29 | |

# Note: The table shows the main compounds whose relative content was above 0.1%

# Supplementary Table 9 Main chemical compositions and relative content of blended-Ⅲ

| NO | Chemical composition | Retention time | Peak area % |
| --- | --- | --- | --- |
| 1 | Linalyl acetate | 19.82 | 19.33 |
| 2 | Limonene | 8.58 | 17.39 |
| 3 | Caryophyllene | 20.88 | 11.95 |
| 4 | Linalool | 19.62 | 11.23 |
| 5 | Pinene | 4.24 | 7.36 |
| 6 | Eucalyptol | 8.88 | 4.77 |
| 7 | β-Pinene | 6.04 | 2.45 |
| 8 | Camphene | 5.07 | 1.88 |
| 9 | γ-Terpinene | 9.99 | 1.81 |
| 10 | Copaene | 17.71 | 1.72 |
| 11 | α-Limonene | 20.59 | 1.65 |
| 12 | Humulene | 23.26 | 1.64 |
| 13 | β-copaene | 24.89 | 1.43 |
| 14 | o-Cymene | 10.72 | 1.42 |
| 15 | α-Terpineol | 24.71 | 1.29 |
| 16 | Geraniol | 33.97 | 1.28 |
| 17 | Camphor | 18.31 | 0.78 |
| 18 | δ-Cadinene | 27.41 | 0.77 |
| 19 | β-Famesene | 23.40 | 0.73 |
| 20 | β-Bisabolene | 25.96 | 0.66 |
| 21 | Geranyl acetate | 27.72 | 0.61 |
| 22 | Lavandulyl acetate | 21.30 | 0.43 |
| 23 | γ-Muurolene | 24.12 | 0.42 |
| 24 | γ-Elemene | 22.24 | 0.41 |
| 25 | β-Elemene | 20.69 | 0.41 |
| 26 | Sabinene | 6.39 | 0.37 |
| 27 | α-Cubebene | 16.69 | 0.37 |
| 28 | Terpinen-4-ol | 21.12 | 0.36 |
| 29 | β-Ocimene | 10.22 | 0.33 |
| 30 | 3-Octanone | 10.29 | 0.32 |
| 31 | trans-β-Ocimene | 9.71 | 0.30 |
| 32 | Caryophyllene oxide | 40.41 | 0.30 |
| 33 | δ-Elemene | 17.07 | 0.25 |
| 34 | β-cyclocitral | 23.81 | 0.20 |
| 35 | Terpinene | 7.99 | 0.18 |
| 36 | γ-Muurolene | 20.19 | 0.17 |
| 37 | 1-Octen-3-yl-acetate | 14.30 | 0.16 |
| 38 | Nerol | 30.68 | 0.15 |
| 39 | Bornyl acetate | 20.41 | 0.15 |
| 40 | β-Selinene | 25.31 | 0.15 |
| 41 | Acetic acid, hexyl ester | 10.90 | 0.14 |
| 42 | Alloaromadendrene | 22.42 | 0.14 |
| 43 | Terpinolene | 11.14 | 0.11 |
| Total | | 97.95 | |

# Note: The table shows the main compounds whose relative content was above 0.1%

**Supplementary Table 10 Correlation between** **gender, region, and fragrance usage habits**

| PCCS | Gender | Region | Fragrance usage habits |
| --- | --- | --- | --- |
| Gender | 1.000 | -0.120 | 0.240 |
| Region | -0.120 | 1.000 | -0.080 |
| Fragrance usage habits | 0.240 | -0.080 | 1.000 |

Pearson’s correlation coefficients (PCCS) were used to assess relationships between gender, region, and fragrance usage habits.

##
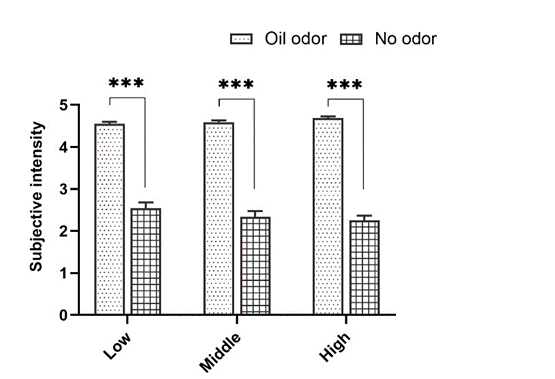
Supplementary Figures

**Supplementary Figure 1.** Effect of intensity on odor sensory evaluation. Mean ratings (±standard error of the mean, n=50) of subjective intensity at low, middle, and high concentrations. ***p< 0.001. T-tests were used to compare the differences between the odor and non-odor groups.
